# Emphysematous Cystitis in a Patient Receiving Cyclophosphamide

**DOI:** 10.7759/cureus.18722

**Published:** 2021-10-12

**Authors:** Said Al Zein

**Affiliations:** 1 Department of Medicine, University of Pittsburgh, Pittsburgh, USA

**Keywords:** immunosuppression, complicated urinary tract infection, rapidly progressive glomerulonephritis, anca-associated vasculitis, cyclophosphamide, emphysematous cystitis

## Abstract

Emphysematous cystitis (EC) is a form of complicated urinary tract infection (UTI) well described in patients with diabetes. Other known risk factors include urinary tract obstruction, older age, and female gender. This case describes a patient who developed emphysematous cystitis while receiving induction therapy with intravenous cyclophosphamide to treat antineutrophil cytoplasmic antibody (ANCA)-associated vasculitis that presented with rapidly progressive glomerulonephritis (RPGN). The association between cyclophosphamide therapy and emphysematous cystitis has only been reported twice in literature.

## Introduction

Emphysematous cystitis (EC) is a form of complicated urinary tract infection (UTI) characterized by air within the bladder wall usually caused by air-forming bacteria [1] and can be life-threatening. Almost two-thirds of patients with EC are diabetics and older females [1]. A recent review of 113 cases of emphysematous cystitis found that other risk factors were neurogenic bladder (9.73%), malignancy on chemotherapy (8.85%), and, even less frequently, immunosuppressed state (transplant recipient) and post-surgery [2]. Only two previous case reports available in the literature have described the association between cyclophosphamide use and EC [3,4].

## Case presentation

An 83-year-old female with a history of hypertension, atrial fibrillation, factor V Leiden mutation, deep vein thrombosis on warfarin, grade 2 cystocele, grade 2 rectocele, and gastroesophageal reflux disease presented to the emergency room with worsening weakness for few days, in addition to dizziness and a lower blood pressure than baseline. She also reported abdominal pain, nausea, and diarrhea. The patient denied any associated dysuria or hematuria. The patient did not report shortness of breath, but her family noticed that she had been tachypneic. Additionally, for 14 weeks prior to presentation, the patient had been receiving induction therapy with intravenous cyclophosphamide infusions and steroids taper for the management of her antineutrophil cytoplasmic antibody (ANCA)-associated vasculitis that manifested as rapidly progressive glomerulonephritis (RPGN) with gross hematuria.

In the emergency room, the patient was alert and oriented and in moderate distress. She had a blood pressure of 107/66 mmHg, a heart rate of 96 beats/minute, a respiratory rate of 18 breaths/minute, a temperature of 97.9°F, and an oxygen saturation on room air of 96%. Her abdomen was soft with mild diffuse tenderness and no guarding or rebound tenderness.

Her most relevant blood tests in the emergency room are summarized in Table 1 and urinalysis with microscopy in Table 2. 

**Table 1:** Blood test results

Test	Result	Reference range
Blood urea nitrogen	58 mg/dL	6–20 mg/dL
Creatinine	1.97 mg/dL	0.5–1.2 mg/dL
Glucose	177 mg/dL	70–99 mg/dL
Potassium	3.8 mg/dL	3.5–5.1 mg/dL
Lactic acid	3.0 mmol/L	0.5–2.2 mmol/L
White blood cell count	8.4 K/uL	4.8–10.8 K/uL
Hemoglobin	9.7 g/dL	12–15.5 g/dL
Platelet count	221 K/uL	150–350 K/uL

 

**Table 2:** Urinalysis

Test	Result	Reference range
Glucose – urine	Negative	Negative
Blood – urine	3+	Negative
Protein – urine	1+	Negative
Urine nitrites	Negative	Negative
Leukocyte esterase	1+	Negative
White blood cell count – urine	15–20 cells/HPF	0–2 cells/HPF
Red blood cells – urine	5–10 cells/HPF	0 cells/HPF

**Figure 1:**
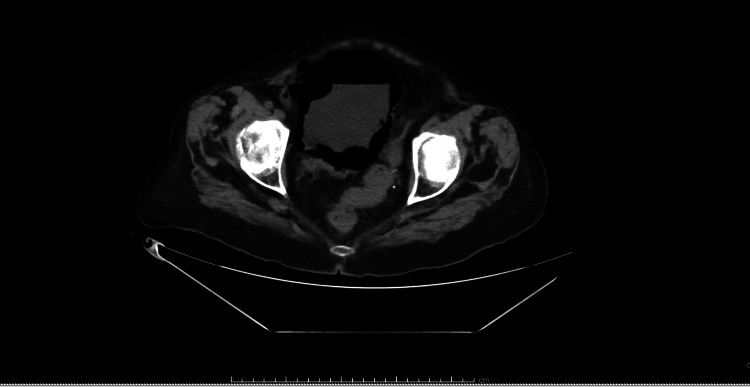
CT scan of the pelvis showing the air within the bladder wall in addition to the air-fluid level indicating air within the bladder lumen

Computed tomography (CT) scan of the abdomen showed emphysematous cystitis with large amount of intraluminal gas in the urinary bladder and gas within the walls of the bladder. There was no gas seen within the pyelocaliceal systems or ureters (Figure 1). The CT also showed chronic appearing interstitial infiltrates in both lung bases. 

The patient was admitted to the hospital and started on ceftriaxone 1 gram intravenously daily and given normal saline solution at 100 mL/hour intravenously for her sepsis. A urinary catheter was not placed at any point during her hospital admission. Her blood cultures were positive for *Escherichia coli* (*E. coli*) in all four bottles and sensitive to most antibiotics including ceftriaxone. Urine culture unfortunately was not obtained upon admission. Two days later, the patient developed a fever of 101°F. However, the repeat blood and urine cultures at that point were all negative. During her hospital stay, the patient was noted to have postprandial hyperglycemia reaching up to 240 mg/dL. Her hemoglobin A1C was 7.8%. Her chart review revealed almost annual hemoglobin A1C results that never exceeded 6% in the past. 

**Figure 2:**
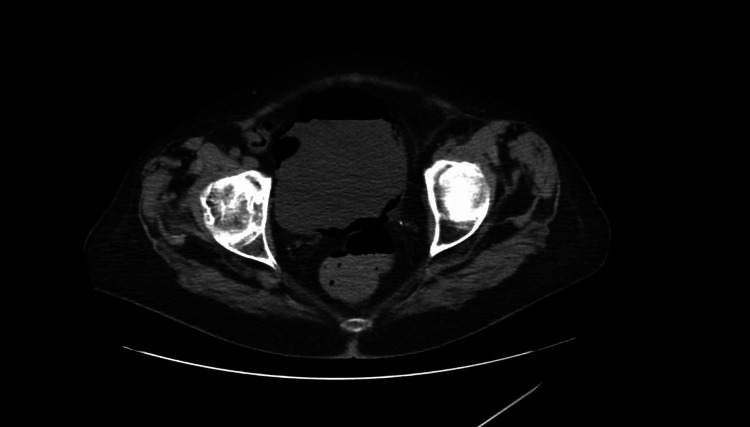
CT scan of the pelvis done after eight days of antibiotics showing improvement in the air within the bladder wall

On day 8 of her admission, the patient had worsening shortness of breath, low-grade fever of 100.9°F, and elevated white blood cell count of 11.9 K/uL (normal range: 4.8-10.8 K/uL). Her urine microscopy did not reveal any white blood cells. Chest X-ray revealed multifocal pneumonia. CT scan showed marked improvement in her emphysematous cystitis (Figure 2). Her antibiotic coverage was switched to vancomycin and piperacillin/tazobactam for the treatment of her hospital-acquired pneumonia. The patient responded well to the pneumonia treatment and was discharged home after one week.

## Discussion

Emphysematous cystitis (EC) is a rare complication of urinary tract infection characterized by air within the bladder lumen or bladder wall usually caused by air-forming bacteria. In a recent review of 113 cases [2], *Escherichia coli* was the most common organism in around 65% of cases, followed by *Klebsiella pneumoniae* (22%). Several other organisms were reported, including *Enterobacter*, *Candida*, and *Citrobacter*. Of the patients with EC, 60% were diabetic, 75% were above 60 years old, and 65% were females. Sepsis and abdominal pain were the most common presenting symptoms. These numbers are comparable with a previous review of 135 cases in 2007 [1].

The patient described in this report was an elderly female. She had never been a diabetic throughout her life but apparently had been having hyperglycemia during her prednisone intake for 14 weeks prior to presentation. Although being a diabetic for less than four months may not lead to the typical chronic complications of diabetes, her hyperglycemia cannot be ignored. Hyperglycemia could have played an important role in the pathogenesis of her EC as some theories suggest that glucose is a substrate for organisms to form carbon dioxide in tissues with high glucose concentration and impaired perfusion [5]. Other theories suggest urinary albumin as the favorable substrate for gas production, which was also present in this patient [6].

The patient had been receiving intravenous cyclophosphamide, an alkylating agent associated with hemorrhagic cystitis, with an incidence of 3.5% when given with mesna [7]. Hemorrhagic cystitis occurs as a complex inflammatory response induced by a toxic metabolite of cyclophosphamide (acrolein) that damages the integrity of the urothelium with swelling, bleeding, and ulceration of the bladder mucosa [8]. This patient did have 5-10 red blood cells/HPF reported on her urine analysis. This was a constant gradual decrease in quantity since the time of her diagnosis with RPGN and initiation of induction therapy. Unfortunately, there was no reliable description of red blood cell morphology to differentiate the glomerular versus non-glomerular etiology of microscopic hematuria.

Another risk factor for EC is bladder outlet obstruction [1]. Our patient had grade 2 cystocele and grade 2 rectocele. The patient did not have any obstructive symptoms and was urinating freely while in the hospital without a urinary catheter.

The management of EC generally consists of antibiotics, bladder drainage, and glycemic control. Surgical intervention with partial cystectomy, cystectomy, or debridement may be needed in patients who do not respond to medical management or those with severe necrotizing infections or bladder perforations [1,2]. Our patient responded well to intravenous ceftriaxone and did not require urinary catheter drainage or any surgical intervention. She had clinical and radiological improvement within a few days. 

## Conclusions

Emphysematous cystitis is increasingly recognized as a complication of cystitis. Detection and appropriate management can prevent fatal complications. More cases are needed to establish an association with cyclophosphamide use.

## References

[1] Thomas AA, Lane BR, Thomas AZ, Remer EM, Campbell SC, Shoskes DA (2007). Emphysematous cystitis: a review of 135 cases. BJU Int.

[2] Ranjan SK, Navriya SC, Kumar S, Mittal A, Bhirud DP (2021). Emphysematous cystitis: a case report and literature review of 113 cases. Urol Ann.

[3] Galloway NT (1984). Gas gangrene of the bladder complicating cyclophosphamide cystitis. Br J Urol.

[4] Abuzarad H, Gadallah MF, Rabb H, Vermess M, Ramirez G (1998). Emphysematous cystitis: possible side-effect of cyclophosphamide therapy. Clin Nephrol.

[5] Huang JJ, Chen KW, Ruaan MK (1991). Mixed acid fermentation of glucose as a mechanism of emphysematous urinary tract infection. J Urol.

[6] Hawtrey CE, Williams JJ, Schmidt JD (1974). Cystitis emphysematosa. Urology.

[7] Almalag HM, Alasmari SS, Alrayes MH (2021). Incidence of hemorrhagic cystitis after cyclophosphamide therapy with or without mesna: a cohort study and comprehensive literature review. J Oncol Pharm Pract.

[8] Brock N, Pohl J, Stekar J (1981). Studies on the urotoxicity of oxazaphosphorine cytostatics and its prevention--I. experimental studies on the urotoxicity of alkylating compounds. Eur J Cancer.

